# Loss of SCRG1 in chondrocytes inhibits osteoarthritis by promoting autophagy activity in the temporomandibular joint through inhibition of neurokine receptors

**DOI:** 10.22514/jofph.2025.020

**Published:** 2025-03-12

**Authors:** JiaJun Zhang, LePing Yuan, YanYan Zhang, HaoYang Jin, YeKe Zhao, XiaoKe Zeng, YanHui Zou, KeYu Wang, Xin Nie

**Affiliations:** ^1^Department of Oral and Maxillofacial Surgery, Hospital of Stomatology Wenzhou Medical University, 325027 Wenzhou, Zhejiang, China; ^2^Department of Oral and Maxillofacial Surgery, The Third People’s Hospital of Yuhang District, 311115 Hangzhou, Zhejiang, China

**Keywords:** TMJOA, SCRG1, Inflammation, Catabolism, Autophagy, NGFR, NF-κB

## Abstract

**Background**: To investigate *in vitro* how scrapie responsive gene 1 (SCRG1) contributes to the development of temporomandibular joint osteoarthritis (TMJOA). **Methods**: Western blotting was used to identify protein expression. Proinflammatory cytokine levels were assessed by means of an enzyme-linked immunosorbent test. In order to find out whether chondrocytes expressed protein light chain 3B (LC3B), immunofluorescence was utilized. **Results**: In the TMJOA *in vitro* model, hydrogen peroxide (H_2_O_2_) treatment increased the expression of SCRG1, stimulated chondrocyte catabolism and inflammatory response, and blocked autophagy. In chondrocytes, SCRG1 silencing reduces the inflammatory response, catabolism, and autophagy inhibition brought on by H_2_O_2_. Concurrently, H_2_O_2_ induction triggers the nuclear factor (NF)-κB pathway and nerve growth factor receptor (NGFR). When SCRG1 is downregulated, NGFR expression is inhibited and the NF-κB pathway is blocked. **Conclusions**: By inhibiting NGFR and blocking the NF-κB pathway, knocking down SCRG1 can prevent H_2_O_2_-induced inflammatory response, metabolic breakdown and autophagy inhibition in chondrocytes.

## 1. Introduction

Millions of people worldwide suffer from temporomandibular joint osteoarthritis (TMJOA), a common and debilitating joint disease that lowers quality of life and causes chronic pain [1]. Throughout life, the temporomandibular joint (TMJ) has active bone remodeling. In early TMJ osteoarthritis, condylar surface erosion, trabecular bone loss, and decreased bone mineral density are the main features of subchondral bone remodeling [2, 3]. Its primary pathological characteristics include joint-wide synovitis, subchondral bone deterioration, and articular cartilage degradation [4]. It is a complex disease with an unclear origin that is marked by a variety of cellular and molecular alterations, including an imbalance in the anabolism and catabolism of cartilage, the infiltration of macrophages, inflammation of the synovium, and immunological reaction activation [5]. Therefore, identifying therapeutic targets and their mechanisms is very important and urgently needed.

Liu and He *et al*. [6] discovered scrapie responsive gene 1 (SCRG1) as a possible therapeutic target for human synovial inflammation by analyzing five Gene Expression Omnibus data sets containing a total of forty-one normal synovial tissues and forty-five synovial samples from osteoarthritis. SCRG1 was identified in 1998 by Dron *et al*. [7] as a gene whose expression was elevated in the brains of mice with scrapie. In a recent work, Dron *et al*. [8] documented the presence of SCRG1 in autophagic vacuoles in end-stage illness and the activation of SCRG1 in the neurons of mice infected with scrapie. Through extracellular signal-regulated kinase (ERK)1/2 activation, SCRG1 prevents lipopolysaccharide (LPS)-induced chemokine-C-C motif chemokine 22 (CCL22) generation in murine macrophage Raw264.7 cells [9]. Mesenchymal stem cells’ capacity for self-renewal, migration, and osteogenic differentiation is controlled by the unique SCRG1/bone marrow stromal cell antigen-1 axis [10]. However, the role and mechanism of SCRG1 in temporomandibular joint osteoarthritis remain unclear and require further clarification.

The purpose of this study was to investigate SCRG1’s function and mechanism in the development of TMJOA. According to our research, SCRG1 knockdown inhibits catabolism and inflammation in the TMJOA model by encouraging autophagy. These findings suggest that SCRG1 may be a useful therapeutic target in the management of TMJOA.

## 2. Method

### 2.1 Isolation and culture of rat chondrocytes

Three-day-old Sprague Dawley (SD) rats’ TMJ were used to isolate primary chondrocytes. The ethical committee gave its approval to the protocol for the animal experiment. The National Institutes of Health, China’s Guide for the Care and Use of Laboratory Animals was followed for approval of all animal-related research projects (Approval No. wydw2023-0661). Following sectioning, the cartilage tissue was processed for 0.5 hours using 0.25% trypsin (T1300, Solarbio, Shanghai, China) including Ethylene Diamine Tetraacetic Acid‌ (EDTA) and for 4 hours using 0.2% collagenase II (ST2303, Beyotime, Shanghai, China). At 37 °C and 5% CO_2_, primary chondrocytes were filtered and suspended in Dulbecco’s Modified Eagle Medium/Nutrient Mixture F (DMEM/F)12 media (D6501, Solarbio, Shanghai, China) supplemented with 1% penicillin-streptomycin and 20% fetal calf serum (C0226, Beyotime, Shanghai, China).

### 2.2 Cell transfection

Using Lipofectamine RNAiMAX (Invitrogen, Carlsbad, CA, USA, 13778-150), transcription knockdown was carried out by transfecting siRNA oligonucleotide duplexes into DMEM for 48 hours at a final concentration of 20 nM. The siRNA oligonucleotide duplex has the following sequence: *SCRG1* (5′-UCUGUGUCAGGUCAGCUACUCCUUC-3; 5′-GAAGGAGUAGCUGACCUGACACAGA-3′).

H_2_O_2_ was added to the growth medium at a 500 µM concentration for both transfected and non-transfected cells. After a whole day, every cell was extracted for additional analysis [11].

### 2.3 Enzyme-linked immunosorbent

The cell supernatant was obtained by collecting the cell culture medium and centrifuging it for five minutes at 500g and 4 °C after treatment. The manufacturer’s instructions were followed while using the interleukin 6 (IL)-6 enzyme-linked immunosorbent assay (ELISA) kit (ab178013, Abcam, Cambridge, UK), Tumour Necrosis Factor alpha (TNF-α) ELISA kit (ab18421, Abcam, Cambridge, UK), and IL-1β ELISA kit (ab179776, Abcam, Cambridge, UK). Levels of interleukin-6 (IL-6) and interleukin-1β (IL-1β) can be manually measured using cell supernatants.

### 2.4 Immunofluorescence

After 10 minutes of 4% paraformaldehyde fixation, 0.1% Triton X-100 permeabilization was applied to the cells. Next, for one hour, cells were blocked with 1% bovine serum albumin (BSA). Following this, cells were tagged with a primary antibody against SCRG1 and LC3B (ab192890, Abcam, Cambridge, UK), and a secondary antibody conjugated with fluorescein was subsequently incubated. Fluorescence microscopy was used to take pictures after nuclei were labeled with 4′,6-diamidino-2-phenylindole (DAPI).

### 2.5 Western blot

Radio Immunoprecipitation Assay (RIPA) was used to extract protein from chondrocytes (R0010, Solarbio Biotech, Shanghai, China). Use sonication to lyse cell lysates for two minutes, then centrifuge at a high speed for fifteen minutes at 4 °C. The protein concentration of bicinchoninic acid (BCA) (PC0020, Solarbio Biotech, Shanghai, China) was then measured by collecting the supernatant. After that, it was heated in a metal bath for ten minutes at 100 °C after being diluted with protein loading buffer (P1040, Solarbio Biotech, Shanghai, China). After separating equal volumes of the extracted proteins using sodium dodecyl sulfate polyacrylamide gel electrophoresis (SDS-PAGE), the proteins were transported to membranes made of polyvinylidene fluoride (PVDF; NIC Biotech, China). After blocking the membrane for one hour at room temperature with 5% skim milk powder, the membrane was incubated for an additional night at 4 °C with certain primary antibodies. After that, the membrane was treated for one hour with the matching horseradish peroxidase-conjugated secondary antibody (7074, CST, Boston, MA, USA). Protein bands were detected using the ChemiDocTM XRS system (PX 2617, Bio-Rad, Heracles, CA, USA) after being contacted with electrochemiluminescence (ECL) solvent (G2014, Servicebio, Wuhan, China). Using glyceraldehyde 3-phosphate dehydrogenase (GAPDH) as the internal reference, ImageJ software was utilized to evaluate and quantify the levels of protein expression. Primary antibodies include SCRG1 (1:1000, ab121384, Abcam, Cambridge, UK), Collagen type X alpha 1 (COL10A1, 1:1000, ab182563, Abcam, Cambridge, UK), aggrecan (ACAN, 1:1000, ab3778, Abcam, Cambridge, UK), matrix metalloprotease-13 (MMP-13, 1:1000, ab315267, Abcam, Cambridge, UK), A Disintegrin and Metalloproteinase with Thrombospondin motifs 5 (ADAMTS5, 1:1000, ab41037, Abcam, Cambridge, UK), protein light chain II/I (LC3II/I, 1:1000, ab232940, Abcam, Cambridge, UK), p62 (1:1000, ab207305, Abcam, Cambridge, UK), p65 (1:1000, ab32536, Abcam, Cambridge, UK), p-p65 (1:1000, ab109458, Abcam, Cambridge, UK), IκB-α (1:1000, ab230341, Abcam, Cambridge, UK), p-IκB-α (1:1000, ab178846, Abcam, Cambridge, UK), Nerve growth factor receptor (NGFR, 1:1000, ab52987, Abcam, Cambridge, UK) and GAPDH (1:3000, ab8245, Abcam, Cambridge, UK).

### 2.6 Statistics

We used GraphPad Prism 9 (GraphPad Software, San Diego, CA, USA) to perform Student’s *t* test (two groups) or one-way Analysis of Variance (ANOVA, more than two groups) to analyze our data, which are expressed as mean ± standard deviation or standard error of the mean. statistical evaluation. Every experiment was conducted at least three times, yielding consistent outcomes. It was deemed statistically significant when *p* < 0.05.

## 3. Result

### 3.1 SCRG1 expression is increased *in vitro* TMJOA model

We took chondrocytes out of the cartilage of the rat knee and temporomandibular joint to investigate the function of SCRG1 in TMJOA. In order to create a more accurate cellular model, we exposed isolated chondrocytes to H_2_O_2_ as an inflammatory agent for a full day. This allowed us to better mimic the pathophysiology of chondrocytes in TMJOA. Based on Western blotting and immunofluorescence analysis, chondrocytes with H_2_O_2_ modeling expressed more SCRG1 than a control group (Fig. 1A,B). These results imply that SCRG1 and TMJOA might be connected.

**Fig. 1.** SCRG1 expression is increased in *in vitro* TMJOA model. (A) Western blotting to detect the expression of SCRG1 before and after H_2_O_2_ modeling. (B) Immunofluorescence detection of SCRG1 fluorescence intensity before and after H_2_O_2_ modeling. Values are presented as mean ± SD. **p < 0.01 versus control group. n = 3. SCRG1: scrapie responsive gene 1; GAPDH: glyceraldehyde 3-phosphate dehydrogenase; H_2_O_2_: hydrogen peroxide.

### 3.2 Knockdown of SCRG1 suppresses inflammation in chondrocytes

Since the expression of SCRG1 is increased in chondrocytes due to H_2_O_2_, we knocked down SCRG1 to explore the role of SCRG1. Western blotting was used to identify silencing (Fig. 2A). To investigate how SCRG1 affects the inflammatory response generated by H_2_O_2_, the levels of inflammatory factors (TNF-α, IL-1β and IL-6) were measured. The findings are displayed in Fig. 2B. TNF-α, IL-1β and IL-6 levels increased in response to H_2_O_2_, whereas SCRG1 silencing decreased these levels. These findings suggest that SCRG1 knockdown reduces the inflammatory response in chondrocytes caused by H_2_O_2_.

**Fig. 2.** Knockdown of SCRG1 suppresses inflammation in chondrocytes. (A) Western blotting to detect protein expression of SCRG1. (B) ELISA detects the levels of IL-6, IL-1β and TNF-α in cell culture medium. Values are presented as mean ± SD. **p < 0.01 versus control group. ^##^*p* < 0.01 versus H_2_O_2_ + si-NC group. n = 3. SCRG1: scrapie responsive gene 1; IL: interleukin; GAPDH: glyceraldehyde 3-phosphate dehydrogenase; H_2_O_2_: hydrogen peroxide; si-NC: si-control; TNF-α: Tumour Necrosis Factor alpha.

### 3.3 Knockdown of SCRG1 inhibits chondrocyte catabolism

*In vitro* tests were conducted to confirm our findings and gain a better understanding of the function of SCRG1 in cartilage homeostasis. Initially, we looked at how SCRG1 affected the *in vitro* chondrocyte catabolism triggered by H_2_O_2_. Proteins associated with cartilage metabolism were examined while chondrocytes were cultivated in a medium enriched with H_2_O_2_. Our findings demonstrate that SCRG1 knockdown efficiently inhibits the rise in MMP-13, COL10A1, and ADAMTS5 levels while preventing the decrease in ACAN brought on by H_2_O_2_ (Fig. 3). When considered collectively, our results imply that SCRG1 knockdown primarily controls cartilage homeostasis by impeding chondrocyte catabolism produced by H_2_O_2_.

**Fig. 3.** Knockdown of SCRG1 inhibits chondrocyte catabolism. Western blotting to detect protein expression of COL10A1, ACAN, MMP-13 and ADAMTS5. Values are presented as mean ± SD. **p < 0.01 versus control group. ^#^*p* < 0.05, ^##^*p* < 0.01 versus H_2_O_2_ + si-NC group. n = 3. SCRG1: scrapie responsive gene 1; COL10A1: Collagen type X alpha 1; ACAN: aggrecan; MMP-13: matrix metalloprotease-13; ADAMTS5: A Disintegrin and Metalloproteinase with Thrombospondin motifs 5; GAPDH: glyceraldehyde 3-phosphate dehydrogenase; H_2_O_2_: hydrogen peroxide; si-NC: si-control.

### 3.4 Knockdown of SCRG1 promotes autophagy in chondrocytes

In this study, we examined the functions of autophagy and SCRG1 in TMJOA *in vitro*. Western blotting and immunofluorescence findings (Fig. 4A,B) demonstrated that H_2_O_2_-induced chondrocytes significantly increased the expression of p62 and significantly decreased the expression of LC3II/I. By destroying SCRG1, the effects of H_2_O_2_ could be reversed (Fig. 4A,B). Research has demonstrated that SCRG1 down-regulation can mitigate the aberrant H_2_O_2_-induced reduction in chondrocyte autophagy.

**Fig. 4.** Knockdown of SCRG1 promotes autophagy in chondrocytes. (A) Western blotting to detect protein expression of LC3II/I and p62. (B) Immunofluorescence detection of fluorescence intensity of LC3B. Values are presented as mean ± SD. **p < 0.01 versus control group. ^##^*p* < 0.01 versus H_2_O_2_ + si-NC group. n = 3. SCRG1: scrapie responsive gene 1; LC3: protein light chain 3; GAPDH: glyceraldehyde 3-phosphate dehydrogenase; H_2_O_2_: hydrogen peroxide; si-NC: si-control.

### 3.5 SCRG1 promotes the expression of NGFR and NF-κB signaling pathways

Prior research has demonstrated that neuroreceptor factor (NGFR) and the NF-κB signaling pathway are important for inflammation and other pathological alterations in OA and TMJOA [12, 13]. We employed Western blotting to identify the expression levels of proteins linked to the NGFR/NF-κB signaling pathway (NGFR, IκB-α, p-IκB-α, p65, and p-p65) in order to investigate whether SCRG1 regulates H_2_O_2_-induced TMJOA through this mechanism. Treatment with H_2_O_2_ markedly enhanced NGFR expression and phosphorylation of p65 and IκB-α. But SCRG1 can be downregulated to lessen these H_2_O_2_-induced alterations (Fig. 5). These results indicate that SCRG1 can promote the expression of NGFR and NF-κB signaling pathways.

**Fig. 5.** SCRG1 promotes the expression of NGFR and NF-κB signaling pathways Western blotting to detect the protein expression of NGFR, p65, p-p65, IκB-α and p-IκB-α. Values are presented as mean ± SD. **p < 0.01 versus control group. ^##^*p* < 0.01 versus H_2_O_2_ + si-NC group. n = 3. SCRG1: scrapie responsive gene 1; NGFR: Nerve growth factor receptor; GAPDH: glyceraldehyde 3-phosphate dehydrogenase; H_2_O_2_: hydrogen peroxide; si-NC: si-control.

## 4. Discussion

Few viable treatment techniques exist for TMJOA due to the uncertain pathophysiology and progression mechanisms of the disease. Persistent pain and swelling severely impair the quality of life for those who have TMJOA [14]. Thus, it is clinically significant to understand the pivotal role that TMJOA development plays and its pathological features. The course of TMJOA is linked to autophagy, and SCRG1 is a crucial regulator of autophagy, inflammatory response, and chondrocyte catabolism. Treatment options for TMJOA may be expanding to include targeting SCRG1 and autophagy. In order to simulate the development of TMJOA *in vitro*, we employed H_2_O_2_ as a stimulant in this work. H_2_O_2_ has the ability to cause inflammatory factor production and chondrocyte death. Therefore, this study employed H_2_O_2_ as a source of free radicals to cause chondrocyte injury in an early model [15].

A protein called SCRG1 is involved in autophagy and has been linked to neurodegeneration in transmissible spongiform encephalopathies and brain damage [16]. Additionally, it was found that SCRG1 is uniquely expressed in human articular cartilage and has a role in the differentiation and growth inhibition of human mesenchymal stem cells (hMSCs) during dexamethasone-dependent chondrogenesis [17]. However, the role of SCRG1 in TMJOA progression has not yet been explored. *In vitro* model of TMJOA, SCRG1 was discovered to be elevated in this study, indicating that SCRG1 might be a target for TMJOA.

Although there is still much to learn about the pathophysiology of TMJOA, inflammation has been identified as a key player. Previous research has shown that increases in pro-inflammatory cytokines are essential to the development of TMJOA [18]. Degradation of cartilage and bone can result from the release of chemokines and enzymes that break down extracellular matrix, which can be triggered by inflammatory stimuli. This procedure may result in TMJOA [19]. Moreover, the chondrogenic development of mesenchymal stem cells from synovial fluid in the temporomandibular joint can be impeded by the production of inflammatory substances [20]. Thus, one possible treatment strategy for TMJOA is to reduce the inflammatory response. According to this study, chondrocytes induced by H_2_O_2_ to secrete inflammatory factors (TNF-α, IL-1β and IL-6) might be inhibited by knocking down SCRG1, which would prevent the inflammatory response.

Matrix metalloproteinase 13 (MMP-13) is the strongest known matrix metalloproteinase that degrades COL2 and is mostly produced by chondrocytes [21]. A significant amount of cartilage matrix is broken down and matrix metalloproteinases are synthesized more often throughout the TMJOA stage. The activity of MMP-13 in chondrocytes rises dramatically as OA progresses [22]. In this work, knocking down SCRG1 increased the production of the chondroprotective factor ACAN and reversed H_2_O_2_-stimulated production of chondrocyte matrix-degrading proteases (including MMP-13 and ADAMTS5) to avoid cartilage erosion.

Autophagy aids in the breakdown and digestion of a cell’s own constituents and preserves the intracellular environment’s dynamic stability. While mild autophagy can prevent cell death and apoptosis, autophagy during times of stress helps cells adapt to changing environmental conditions and guarantees the acquisition of substances necessary for the synthesis of macromolecules for survival [23]. Autophagy plays a key role in the development and incidence of many diseases, including cancers, liver, renal, and cerebrovascular illnesses. It is conceivable that this process will develop into a new therapeutic entry point as the illness progresses [24]. Autophagy-related indicators such LC3-II have markedly elevated gene expression levels in chondrocytes during the early stages of OA. As the illness worsens, autophagy gradually declines and oxidative stress-induced damage gradually increases, resulting in chondrocyte hypertrophy [25]. On the other hand, autophagy plays a significant role in controlling immunological responses. It has the ability to control inflammatory reactions in addition to eliminating infectious sources [26]. As Yang’s research shows, DDIT3-inhibited autophagy plays a crucial role in condylar cartilage degeneration during TMJOA development [27]. This work shows that the suppression of autophagy in chondrocytes caused by H_2_O_2_ can be reversed by knocking down SCRG1. These findings suggest a potential connection between SCRG1 and autophagy in the pathogenic development of TMJOA.

Although our study has elucidated the role of SCRG1, its exact mechanism of action remains to be investigated. Through the osteochondral junction, sensory and sympathetic nerves penetrate the cartilage in TMJOA after inflammation and angiogenesis [28]. NGFR is increased in TMJOA cartilage, according to studies. This factor induces pain by changing the pain threshold by increasing the proliferation of sensory neurons [29]. Furthermore, a growing body of research indicates that NF-κB signaling plays a crucial role in the development of TMJOA. Furthermore, the NF-κB axis is a significant signaling system that controls inflammation-induced damage in number of different disorders [30, 31]. In the TMJOA *in vitro* model used in our work, NGFR and NF-κB pathway was activated. This result was reversed by knocking down SCRG1.

This research still has certain limitations. The results have not been verified in animal models and clinical experiments, and the mechanism research is too superficial. Present results seem novel and promising which encourage to conduct more in-depth research in the future.

## 5. Conclusions

In conclusion, we discovered that the TMJOA *in vitro* model had an elevated level of SCRG1. By encouraging NGFR and blocking the NF-κB pathway, knocking down SCRG1 can prevent the inflammatory response, metabolic breakdown, and autophagy inhibition of chondrocytes brought on by H_2_O_2_. According to this study, TMJOA may benefit from treatment that targets SCRG1.
